# Obituary

**Published:** 1890-01

**Authors:** 


### Obituary.

Died in Dayton, Ohio, on Nov. 29th, of Pneumonia, Dr. Wm. A. Pease, M.D., D.D.S., aged 71 years.

Dr. Pease was born in Norfolk, Conn., in 1818. After taking a thorough course in preparation, he practiced medicine in New York from 1847 to 1849. From an inclination to follow a specialty he, about the latter period, took up the practice of dentistry. He selected Dayton, Ohio, as the field for his future work, where he remained till his death. He was in the active practice of his profession for over thirty-five years, when from failing health, he had, in a measure, to relinquish his work, nevertheless such was his attachment to it that he continued to persue it, as his health would allow, on till the time of his last illness.

Dr. Pease was a man of more than ordinary endowments and education; had a high sense of professional honor and integrity; he had the utmost contempt for quackery in all its phases, he had no patience with the mere pretender, but was most confiding in those whom he regarded as true and honest. He was for many years in the enjoyment of a large and lucretive practice, so that he was at one time in very good circumstances financially, but through misfortune, in which the dishonesty of others played an important part, it passed out of his possession. and for several years he was made to feel what it was to be in straightened circumstances; and this had somewhat of a depressing influence upon him, still he was disposed to make the most of what he had.

He was a man of many good qualities which were well worthy of emulation, which might well be imitated by all. He will be remembered by all who knew him with kind regard and high respect.

### Obituary.

Died, of typhoid pneumonia, Thursday, January 2, Dr. H. L. Moore of Cincinnati. Dr. Moore was a graduate of the Ohio College of Dental Surgery, of the class of 1879; and has practiced his profession since that time in this city. He was one of the rising and promising men of the dental profession in this part of Ohio; a man of true professional honor and purpose; he had the high regard and esteem of all who knew him. He was interested in all that would promote the welfare of his chosen calling, and was not only interested, but active in every good word and work. The profession can ill afford to loose such members. He will not only be missed from his profession, but by his neighbors and friends as well; he was a public spirited citizen, and an exemplary Christian gentleman. The bereaved family will have the warmest sympathy of all his friends and acquaintances.

